# Development of a candidate stabilizing formulation for bulk storage of a double mutant heat labile toxin (dmLT) protein based adjuvant

**DOI:** 10.1016/j.vaccine.2017.03.101

**Published:** 2017-10-04

**Authors:** Vishal M. Toprani, Neha Sahni, John M. Hickey, George A. Robertson, C. Russell Middaugh, Sangeeta B. Joshi, David B. Volkin

**Affiliations:** a Department of Pharmaceutical Chemistry, Macromolecule and Vaccine Stabilization Center, University of Kansas, 2030 Becker Drive, Lawrence, KS 66047, USA; b The Center for Vaccine Innovation and Access, PATH, 455 Massachusetts Ave NW Suite 1000, Washington, DC 20001, USA

**Keywords:** Adjuvant, dmLT, Formulation, Stability, Excipient, Aggregation

## Abstract

This work describes the formulation design and development of a novel protein based adjuvant, a double mutant of heat labile toxin (dmLT), based on knowledge of the protein’s structural integrity and physicochemical degradation pathways. Various classes of pharmaceutical excipients were screened for their stabilizing effect on dmLT during exposure to thermal and agitation stresses as monitored by high throughput analytical assays for dmLT degradation. Sucrose, phosphate, sodium chloride, methionine and polysorbate-80 were identified as potential stabilizers that protected dmLT against either conformational destabilization, aggregation/particle formation or chemical degradation (e.g., Met oxidation and Lys glycation). Different combinations and concentrations of the selected stabilizers were then evaluated to further optimize dmLT stability while maintaining pharmaceutically acceptable ranges of solution pH and osmolality. The effect of multiple freeze-thaw (FT) cycles on the physical stability of candidate bulk formulations was also examined. Increasing the polysorbate-80 concentration to 0.1% in the lead candidate bulk formulation mitigated the loss of protein mass during FT. This formulation development study enabled the design of a new bulk formulation of the dmLT adjuvant and provides flexibility for future use in combination with a variety of different vaccine dosage forms with different antigens.

## 1 Introduction

Adjuvants are molecules/agents, that when properly formulated with certain antigen(s) in a pharmaceutical vaccine dosage form, enhance the desired immune response to the antigen(s) including elevated antibody or cellular responses, improved duration of vaccine protection, and/or reducing the required dose of an antigen [1], [2], [3]. Although there are several adjuvants approved for use with specific antigens (e.g., aluminum salts, oil-in-water emulsions, and the immune stimulating molecule monophosphoryl lipid A, MPL), in the case of newer adjuvants, only a handful have advanced from preclinical stages into clinical testing [4], [5]. The reasons for the failure of such novel adjuvants during the early stages of development is not only undesirable biological activity or side effects, but from a pharmaceutical perspective, poor compatibility with antigens and/or instability during long-term storage leading to loss of potency [6]. The need for systematic formulation development studies, which can be used to minimize instability and maintain/augment the potency of adjuvants, is often poorly recognized in the field of vaccinology [4]. For example, *in vivo* animal studies are often used to monitor adjuvant structural integrity, while, sensitive biophysical and analytical assays can often help to better understand the structural characteristics and the instabilities of adjuvants and link them to the key critical quality attributes of a vaccine such as potency [7].

Since vaccine dosage forms are multicomponent and inherently complex in nature (e.g., biological antigens, adjuvants, excipients), it becomes essential as part of their development to study the factors that can affect their stability and potency. Recombinant or inactivated vaccine antigens, such as proteins, virus-like particles, and inactivated viruses, are often inherently marginally stable and are sensitive to external conditions such as changes in pH and temperature, surface interactions and agitation, light exposure, and even the presence of impurities from excipients [8]. For example, vaccines may be exposed to temperature fluctuations during manufacturing, storage, and transport from the manufacturer to the end user (e.g., clinic site) [9]. The fill finish process can induce mechanical stress on the protein antigen or adjuvant causing aggregation and structural alterations [10], [11]. In addition, the lack of a robust cold chain in developing countries [12] can expose vaccines to elevated temperatures, as well as freeze thaw effects, causing degradation and loss of potency.

To design a stable and efficacious vaccine for clinical use, formulation development activities include preformulation characterization of the antigen including forced degradation studies to identify stability indicating assays, formulation design and excipient screening to define stabilizing solution conditions, and accelerated and real time stability studies as well as freeze-thaw studies [7], [13] to ensure that the candidate formulations remain stable and maintain potency over a defined shelf life. Pharmaceutical excipients are added not only to stabilize the vaccine antigen, but also to ensure appropriate interaction with the adjuvant (e.g., binding of antigen to aluminum adjuvant) as well as to maintain appropriate solubility, tonicity, and compatibility with containers and administration procedures [13], [14], [15], [16].

The physicochemical stability profile of an early stage, lyophilized formulation of dmLT (Toprani et al., 2017, submitted) shows the protein is prone to several physicochemical instabilities both during processing and after reconstitution. For example, dmLT was prone to certain levels of Lys glycation by lactose and formation of small amounts of aggregates upon reconstitution from the lyophilized state. Upon reconstitution, under forced degradation conditions, physical instability of dmLT (A-subunit) upon heating, aggregation during agitation, and chemical instability (Asn deamidation and Met oxidation) upon changes in pH/addition of oxidants were observed. In this work, we seek to utilize the knowledge gained from the structural characterization and elucidation of physicochemical degradation pathways of dmLT to develop a more stable bulk formulation of dmLT that does not require lyophilization and permits maximal flexibility for use in the future in different vaccine dosage forms with different antigens.

## 2 Materials and methods

Lyophilized vials of dmLT, produced and purified from *E. coli* as described elsewhere [17], were received from Walter Reed Army Institute of Research, MD, (Batch No: BPR-1037.00, 21 Nov 2011) and stored at −20 °C. Freeze-dried samples contained 0.7 mg protein in 42.7 mM sodium phosphate, 10.7 mM potassium phosphate, 82 mM NaCl, 5% lactose, pH 7.4. The lyophilized vials (0.7 mg dmLT/vial) were reconstituted in 0.7 mL of HPLC grade water (Fisher Scientific, PA) prior to analysis. Dialysis of dmLT protein for excipient screening was performed in Slide-A-Lyzer Mini Dialysis Devices (Product #88403, Thermo Scientific, Rockford, IL) with a 3500 Da molecular weight cutoff. All reagents and excipients for preparing different formulations were purchased from Sigma-Aldrich (St. Louis, MO).Carbohydrates such as trehalose, sucrose and mannitol were purchased from Pfanstiehl Inc. (Waukegan, IL). All excipients were of high purity grade (>99%).

For a detailed description of analytical methods, including optical density at 350 nm, micro-flow imaging, differential scanning calorimetry, UV–visible absorption spectroscopy, intact mass spectrometry, see the supplemental method section. The sample preparation for excipient screening, agitation, thermal, freeze thaw and forced oxidation and glycation studies can also be found in supplemental method section.

## 3 Results

### 3.1 Screening of pharmaceutical excipients to improve physical stability of dmLT

Since thermal and agitation induced aggregation was identified as a major physical degradation pathway for dmLT (Toprani et al., 2017, submitted), assays used to monitor dmLT aggregation were used to identify stabilizing conditions and additives as a first step to design an optimized formulation. To permit effective screening of excipients that minimize thermal (heat) and agitation induced aggregation, high throughput stability-indicating assays such as increases in optical density at 350 nm (during heating) and micro-flow imaging particle counting (after agitation) were implemented. By starting the excipient screening with the goal of minimizing aggregation, we subsequently identified conditions/excipients that would also minimize the chemical degradation pathways such as Asn deamidation, Lys glycation and Met oxidation. A base buffer of 10 mM sodium phosphate, 150 mM NaCl, pH 6.0 was chosen for these initial excipient screening studies since this solution pH resulted in suboptimal dmLT physical stability (based on biophysical studies described elsewhere; Toprani et al., 2017, submitted), allowing for efficient screening of excipient’s ability to improve dmLT stability. Ten different categories of pharmaceutical excipients were evaluated including carbohydrates, polyols, amino acids, carboxylic acids, salts, metal ions/chelators, detergents, cyclodextrins, polymers/proteins, and polyions/osmolytes (see Supplemental Table S1 for complete list of excipients examined in this work).

**Fig. 1:**
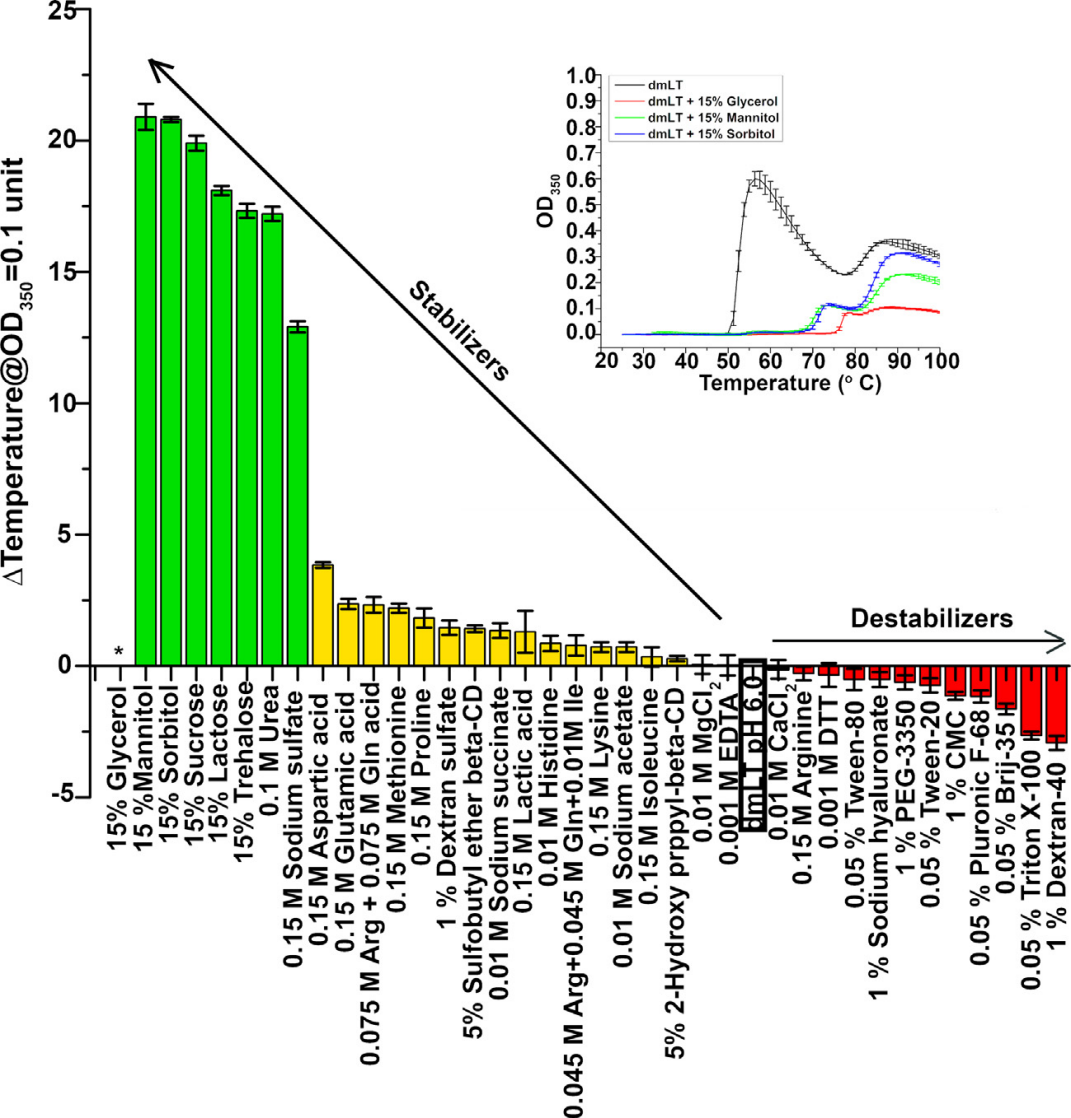
OD_350_ studies of aggregation propensity (delta temperature values) of dmLT containing solutions after thermal stress in presence of different excipients. Average delta temperature value at which the OD_350_ value reaches 0.1 absorbance unit for dmLT (0.15 mg/mL) in control buffer plus excipient vs. dmLT in control buffer alone (10 mM sodium phosphate, 150 mM NaCl, pH 6.0 with no additional excipient; highlighted box). The dmLT samples are shown in order of highest to lowest OD_350_ (indicating highest to lowest stability in terms of aggregation behavior). Error bars represent the standard deviation from triplicate experiments. The inset shows the representative OD_350_ thermal melt experiment of dmLT formulated in control buffer alone and control buffer in the presence of glycerol, mannitol and sorbitol. Excipients in green, yellow and red showed a large increase, moderate increase and low/no increase in stability, respectively. ^*^For 15% glycerol, OD_350_ value did not reached 0.1 absorbance unit and hence delta temperature could not be calculated.

**Fig. 2:**
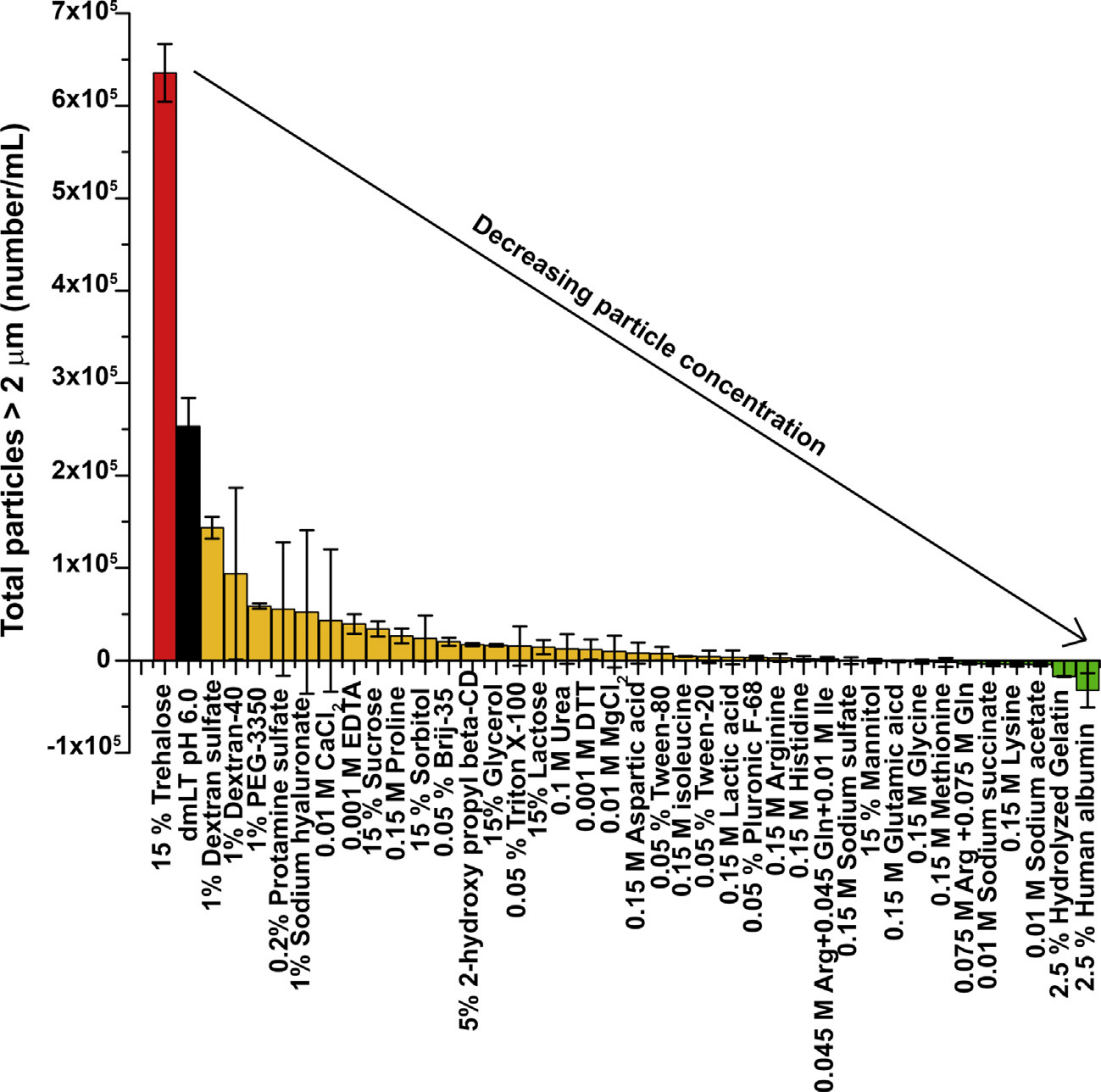
MFI studies of subvisible particle formation in dmLT containing solutions after agitation stress as a function of excipient addition. Total increase in the sub-visible particle concentration (after 4 h of agitation minus time zero results from the same solution) is shown for each of the dmLT samples in order of decreasing particle concentration. The control (dmLT (0.15 mg/mL) in 10 mM sodium phosphate, 150 mM NaCl, pH 6.0 with no additional excipient) is indicated by black bar and is included for reference. Error bars represent the standard deviation from triplicate experiments. Excipients added to dmLT in control buffer in green, yellow and red showed a large increase, moderate increase and low/no increase in number of sub-visible particles, respectively.

Fig. 1 shows the effect of excipients on the aggregation propensity of dmLT during thermal stress. The average delta temperature value to reach 0.1 OD_350_ unit in the dmLT control formulation plus excipient *vs.* dmLT control formulation alone (10 mM sodium phosphate, 150 mM NaCl, pH 6.0) were sorted from highest to lowest values, indicating highest to lowest stability of dmLT in terms of aggregation behavior. Carbohydrates and polyols including glycerol, sucrose, mannitol, trehalose, sorbitol and lactose showed a larger stabilizing effect towards minimizing dmLT aggregation compared to other excipients such as amino acids and detergents, which ranged from moderate to low/no increase in dmLT stability against aggregation. The effect of these additives on the aggregation propensity of dmLT during agitation (shaking of vials containing dmLT in different solutions) as measured by MFI is shown in Fig. 2, where the excipients added to the control formulation were sorted from highest to lowest values of total sub-visible particle concentration. With the exception of trehalose, the sub-visible particle concentration of the dmLT solutions after shaking was lower with each of the tested excipient conditions compared to the control formulation alone. Excipients such as hydrolyzed gelatin, human albumin and sodium acetate were found to be the most stabilizing against shaking induced aggregation. However, since albumin and gelatin are proteins, their effect on dmLT aggregation in OD350 assay could not be evaluated since the additives themselves may aggregate. Sodium acetate was not further investigated due to its minimal effect on thermally induced aggregation as compared to carbohydrates. A tabular summary of the results from OD_350_ measurements (thermal stress, Fig. 1) and total sub-visible particle concentration from MFI measurements (agitation stress, Fig. 2) for the dmLT samples in the presence of various excipients are also summarized in Supplemental Table S1.

Different concentrations of top performing aggregation inhibitors from four different excipient categories (sugars, amino acids, metal chelators and detergents) were then selected and further evaluated to explore the concentration dependence of their stabilizing effect on dmLT using both thermal and agitation stresses (Table 1). Higher concentrations of polyols and sugars resulted in a higher dmLT thermal stability as seen by increased delta temperature values from the OD350 assay. Several different amino acids exhibited a slight positive concentration effect by increasing the thermal stability of dmLT while chelators/detergents did not have a detectable effect. For agitation induced aggregation of dmLT monitored by MFI, all of the tested concentrations of stabilizers had a protective effect against shaking induced destabilization of dmLT measured by the total number of sub-visible particles. Amino acids and polysorbate 20/80 appeared to be the most promising excipients in limiting sub-visible particle formation during agitation stress.

**Table 1:** Concentration optimization of lead stabilizing excipients for their stabilizing effect on dmLT (0.15 mg/mL) aggregation due to thermal stress as measured by OD_350_ thermal melt and agitation stress as monitored by MFI. All excipients were formulated in 10 mM sodium phosphate, 150 mM NaCl, pH 6.0. Data represents mean and standard deviation of three replicates. NA indicates not applicable.

Excipient	Concentration	Thermal stress		Agitation stress	
		Δ Temperature@OD_350_ = 0.1 unit (°C)		MFI (ΔTotal particles_4h–0h_ > 2 µm, number/mL)	
		Mean	SD	Mean	SD
Glycerol	5% w/v	3.8	0.2	96,360	475
	7.5% w/v	20.0	0.2	41,429	9753
	10% w/v	23.0	0.5	3956	2063
	15% w/v	NA	NA	16,228	1311
Sorbitol	5% w/v	3.5	0.2	49,199	11,559
	7.5% w/v	16.1	0.1	92,553	602
	10% w/v	18.0	0.2	83,439	7221
	15% w/v	20.8	0.1	23,767	2478
Mannitol	5% w/v	2.8	0.2	1105	1055
	7.5% w/v	14.4	0.8	433	791
	10% w/v	15.8	0.2	5375	2743
	15% w/v	20.9	0.5	−702	2198
Sucrose	5% w/v	2.0	0.2	−2419	127
	7.5% w/v	3.1	0.5	−336	538
	10% w/v	3.6	0.4	4322	533
	15% w/v	19.9	0.3	33,934	8183
Lactose	5% w/v	3.3	0.3	717	117
	7.5% w/v	13.4	0.2	15,616	4412
	10% w/v	15.5	0.2	36,470	14,092
	15% w/v	18.1	0.2	14,196	7817
Aspartic acid	25 mM	0.1	0.2	9017	3617
	75 mM	1.2	0.1	7718	4069
	150 mM	3.9	0.1	7927	11,322
Methionine	25 mM	−0.2	0.1	4494	3747
	75 mM	0.1	0.1	6255	6446
	150 mM	2.2	0.2	−2224	4911
Arginine	25 mM	0.5	0.2	−1794	602
	75 mM	−0.1	0.2	−3523	1861
	150 mM	−0.2	0.2	2672	4301
Histidine	25 mM	0.6	0.2	22,812	3262
	75 mM	0.7	0.2	994	258
	150 mM	0.7	0.2	1657	2920
EDTA	0.05 mM	−0.3	0.6	−4672	1168
	0.1 mM	−0.5	0.5	8195	691
	1 mM	0.4	0.1	39,368	10,677
Polysorbate 80	0.01% v/v	−0.3	0.4	−9167	2773
	0.025% v/v	−0.7	0.0	−10,018	495
	0.05% v/v	−0.4	0.3	7241	7172
Polysorbate 20	0.01% v/v	−0.5	0.1	15,616	1794
	0.025% v/v	−0.8	0.2	−12,947	5294
	0.05% v/v	−0.6	0.1	3881	6758

Based on these results, only a selected number of the most promising excipients were chosen for further combination screening based on their demonstrated stabilizing effect on dmLT (Table 1) as well as practical considerations from a formulation development perspective such as their effect on solution osmolality. For example, for the selection of sucrose, even though many of polyols/sugars (such as glycerol, sorbitol, mannitol and lactose) inhibited thermally induced aggregation of dmLT to a greater extent than sucrose, these additives were not selected for further optimization. Glycerol and mannitol solutions of equal w/v ratio have higher osmolality values compared to sucrose solutions. When sucrose and glycerol are compared at equivalent osmolality (10% sucrose vs 5% glycerol), sucrose is equally effective at inhibiting heat-induced aggregation of dmLT, and in addition, resulted in a lower number of sub-visible particles formed during agitation. Similarly, sorbitol and lactose solutions also showed higher number of sub-visible particle formation than sucrose solutions during agitation. Moreover, sorbitol and mannitol may manifest a lesser ability to protect during freezing and thawing compared to disaccharides at equivalent solution osmolality values [18], [19], [20], [21], a consideration of importance for dmLT as described below. Finally, lactose is a reducing sugar which can glycate Lys residues in proteins (as was observed with dmLT, Toprani et al., 2017, submitted), a reaction that can potentially lead to protein instability as well as formation of advanced glycation products [22], [23]. As shown below, addition of sucrose in the formulation does inhibit glycation of dmLT, compared to lactose, under forced degradation conditions of elevated temperature. Based on these considerations, 10% sucrose was selected as a sugar-based stabilizer to improve overall stability of dmLT against aggregation without the concern of causing protein glycation.

In addition, polysorbate-80 and methionine were the other two stabilizers selected for further work to develop a stabilizing formulation of dmLT (Table 1). Polysorbate-80 greatly minimized agitation induced aggregation of dmLT (despite its mild destabilizing effect on thermally induced aggregation). Methionine showed some stabilization against agitation induced aggregation (and no destabilization against thermally induced aggregation) of dmLT (Table 1), yet was primarily selected for its ability to mitigate the potential of oxidative stress (oxidation was identified as major chemical degradant pathway of dmLT as shown during forced oxidation studies; Toprani et al., 2017, submitted). As shown below, addition of Met in the formulation does inhibit hydrogen peroxide induced oxidation of Met residues in dmLT.

### 3.2 Salt optimization for dmLT stability

**Fig. 3:**
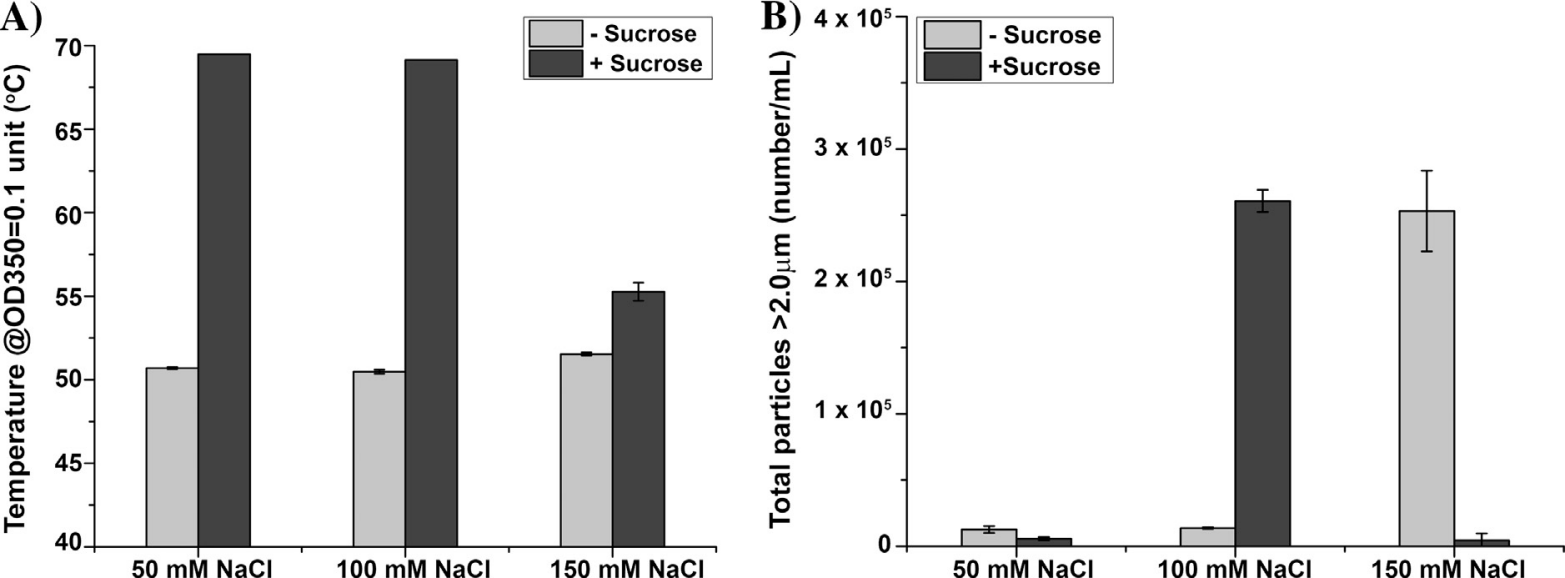
Effect of sodium chloride concentration on dmLT physical stability profile at 0.15 mg/mL in a base buffer containing 10 mM phosphate buffer, ±10% w/v sucrose, pH 6.0. (A) Thermal stress as monitored by OD_350_ temperature values of dmLT as a function of salt concentration. Average temperature value at which the OD_350_ value reaches 0.1 of different concentrations of salts is shown, and (B) agitation stress as measured by MFI in terms of total increase in sub-visible particle concentration (agitation for 4 h minus time zero for same solution) is shown for each of the dmLT samples. Error bars represent the standard deviation from triplicate experiments.

Sodium chloride was observed to be essential to maintain the solubility of dmLT in solution during dialysis. Higher sodium chloride concentrations in combination with other excipients, however, can lead to high solution osmolality which may be undesirable for clinical administration. Therefore, to balance sufficient dmLT solubility with total solution osmolality values, different concentrations of sodium chloride (50, 100, and 150 mM) in 10 mM sodium phosphate buffer pH 6.0 (±10% w/v sucrose) were tested to identify the salt concentration-dependence of the stabilizing effect on dmLT. As shown in Fig. 3A, in the absence of sucrose, increasing NaCl concentration had no major effect on the thermal stability of dmLT as measured by the OD_350_ thermal stress assay_._ In the presence of sucrose, however, dmLT formulated with 50 and 100 mM NaCl showed higher thermal stability in comparison to 150 mM NaCl. In terms of agitation stress as shown in Fig. 3B, MFI results showed that the 50 mM sodium chloride containing solutions of dmLT produced a lower number of sub-visible particles compared to 100 and 150 mM sodium chloride. These results demonstrated that, in general, dmLT samples formulated with sucrose were thermally more stable toward aggregation compared to samples without sucrose, and a combination of sucrose (10% w/v) and 50 mM NaCl limited aggregation and particle formation caused by agitation stress. Finally, dmLT formulated in the presence of 10% w/v sucrose and 50 mM sodium chloride at pH 6.0 had lower solution osmolality than formulations containing higher sodium chloride concentrations (∼450 mOsm for 50 mM NaCl vs ∼650 mOsm for 150 mM NaCl). Based on these results, 10 mM sodium phosphate, 50 mM NaCl, 10% w/v sucrose, pH 6.0 was selected as the base buffer for further optimization of dmLT physical stability in combination with other promising excipients such as methionine and polysorbate-80.

### 3.3 Identifying optimal combinations of lead stabilizers

Combinations of the lead stabilizers were tested to determine any potential additive or synergistic effects on dmLT stability and to check for incompatibility between excipients. Based on the considerations outlined above, a candidate formulation including sodium phosphate buffer, sodium chloride, sucrose, methionine and PS-80 was identified which showed protection against aggregation induced by thermal and agitation stresses, and potentially could help prevent oxidation in dmLT.

**Fig. 4:**
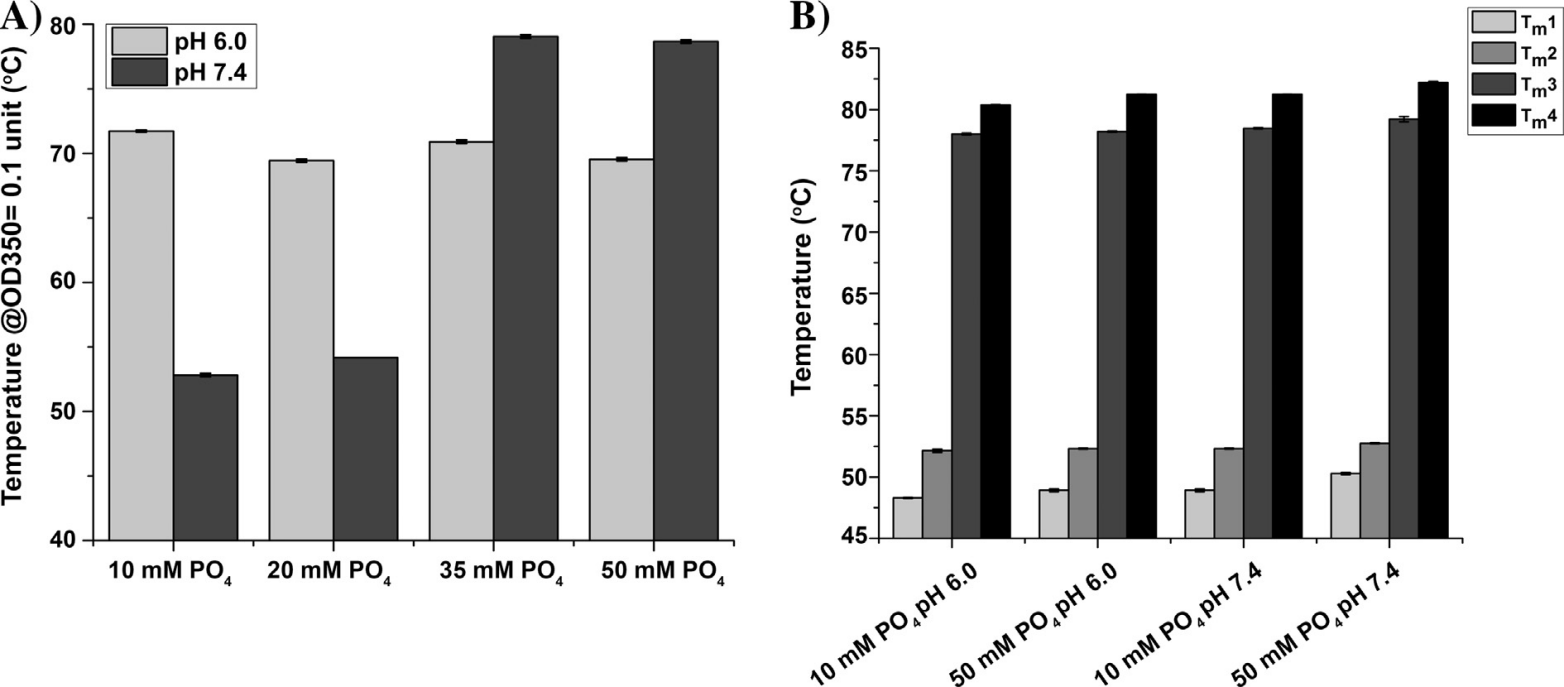
Effect of phosphate buffer concentration and pH on the thermal stability of dmLT at pH 6.0 and pH 7.4 in two different candidate formulations (see text for formulation conditions). (A) Temperature to reach 0.1 absorbance unit as measured by OD_350_ thermal melts assay with dmLT in different solutions, and (B) Tm values for dmLT in two different candidate formulations (pH 6.0 and pH 7.4) containing an additional 10 and 50 mM phosphate ion as measured by DSC. Error bars represent the standard deviation from triplicate experiments.

The effect of both solution pH and phosphate concentration on the physical stability of dmLT in the candidate formulation was first evaluated. Briefly, 10, 20, 35 and 50 mM phosphate at both pH 6.0 and 7.4 were evaluated (in combination with 50 mM NaCl, 10% sucrose, 5 mM methionine and 0.01% v/v PS-80). The phosphate concentration did not appear to affect the thermal stability of dmLT at pH 6.0 (Fig. 4A) as measured by the OD350 assay. Interestingly at pH 7.4, however, an increase in phosphate concentration increased the thermal stability of dmLT as measured by the same OD350 assay. To further understand these results, differential scanning calorimetry was employed to assess the effect of the higher phosphate concentration on the overall conformational stability of dmLT at the two different pH values. Fig. 4B shows the thermal melting temperature (Tm) values for dmLT formulated in both 10 and 50 mM phosphate at both pH 6.0 and 7.4 (in combination with 50 mM NaCl, 10% sucrose, 5 mM methionine and 0.01% v/v PS-80). No major differences were observed in the Tm values of dmLT formulated in 10 vs. 50 mM phosphate at pH 6.0. However, dmLT formulated in 50 mM phosphate at pH 7.4 had slightly higher Tm values compared to 10 mM phosphate pH 7.4 (a ∼2 °C increase in Tm1, corresponding to unfolding of the A-chain of dmLT). These studies showed two different pathways of excipient stabilization of dmLT at the two different pH conditions, pH 6.0 and 7.4 (see discussion section below). At pH 6.0, the presence of sucrose had a major stabilizing effect on dmLT, while phosphate concentration did not affect thermal stability. Conversely at pH 7.4, a higher phosphate concentration showed higher dmLT thermal stability. Based on these results, two candidate dmLT formulations were identified for additional evaluations (1) 10 mM sodium phosphate (pH 6.0), 50 mM NaCl, 10% w/v sucrose, 5 mM methionine, 0.01% v/v PS-80, and (2) 50 mM sodium phosphate (pH 7.4), 50 mM NaCl, 10% w/v sucrose, 5 mM methionine, 0.01% v/v PS-80.

**Supplementary Figure 1:**
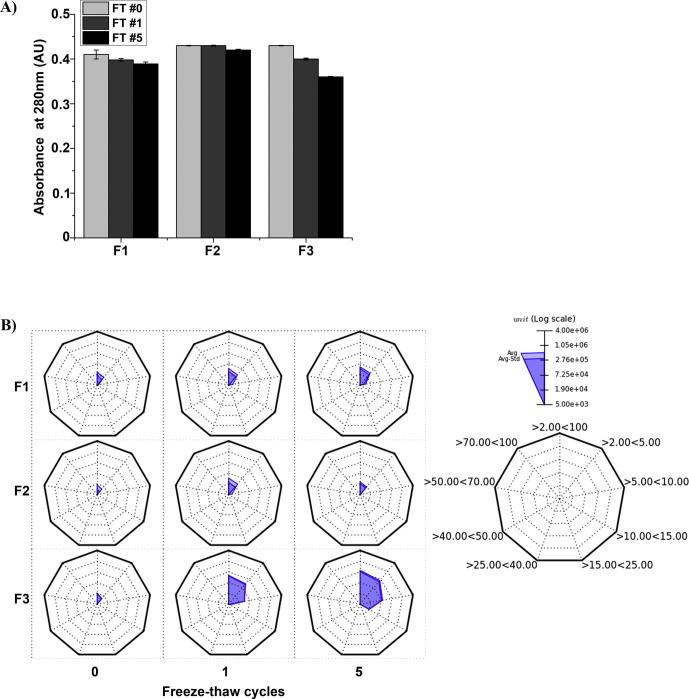


The effect of multiple freeze-thaw (F/T) cycles (0, 1 and 5 cycles) on dmLT in these two candidate liquid formulations, along with a comparison to the current dmLT formulation used to prepare the lyophilized dosage form, was evaluated using UV–Visible spectroscopy to monitor protein concentration (and aggregation) as well as MFI to measure subvisible particle formation. The current lyophilized dmLT formulation showed a higher protein loss (∼17%) as compared to the two candidate formulations (∼6%) after five F/T cycles (Supplemental Fig. S1A). The current formulation also showed a greater number of sub-visible particles/aggregates in comparison to both the candidate formulations after five F/T cycles. Between the two candidate formulations, the pH 6.0 formulation showed higher numbers of particles than the pH 7.4 formulation after five F/T cycles (Supplemental Fig. S1B).

**Fig. 5:**
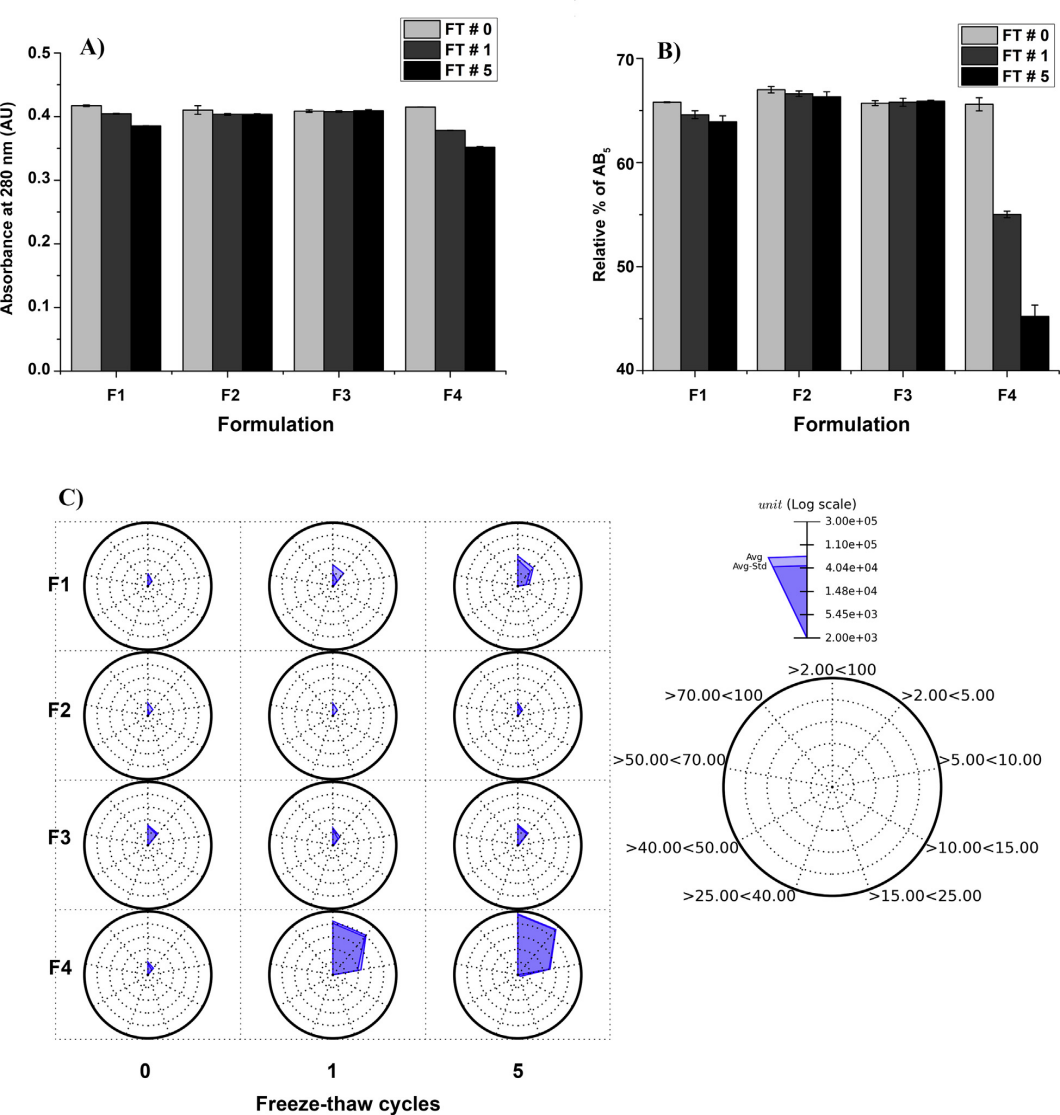
Effect of PS-80 concentration (0.01, 0.05 and 0.1% w/v) on freeze-thaw (0, 1 and 5 freeze-thaw cycles) stability of dmLT compared to dmLT in the current formulation. (A) Absorbance at 280 nm showing protein loss with increasing freeze thaw cycles, (B) % of native dmLT (AB_5_ complex) as a function of freeze-thaw cycles as measured by HIC, and (C) radar plot analysis of the number and size distribution of sub-visible particles formed upon freeze-thaw as measured by MFI. The dmLT protein concentration was 0.4 mg/mL in the four formulations namely F1: 50 mM sodium phosphate, 50 mM NaCl, 10% w/v sucrose, 5 mM methionine, 0.01% v/v PS-80 pH 7.4; F2: 50 mM sodium phosphate, 50 mM NaCl, 10% w/v sucrose, 5 mM methionine, 0.05% v/v PS-80 pH 7.4; F3: 50 mM sodium phosphate, 50 mM NaCl, 10% w/v sucrose, 5 mM methionine, 0.1% v/v PS-80 pH 7.4 and F4: 42.7 mM sodium phosphate, 10.7 mM potassium phosphate, 82 mM NaCl, 5% lactose, pH7.4 (current formulation buffer). Error bars indicate standard deviation of triplicate samples.

Based upon these results, the candidate formulation at pH 7.4 was further optimized to limit protein loss during multiple F/T cycles. The loss of protein during F/T in the candidate formulation at pH 7.4 was minimized by increasing the PS-80 concentration from 0.01% v/v PS-80 to 0.05 and 0.1% v/v PS-80 as shown in Fig. 5. The effect of increasing PS-80 concentration in the candidate formulation on the freeze-thaw stability of dmLT was monitored by a combination UV–Vis spectroscopy (protein loss), hydrophobic interaction chromatography (HIC, to measure relative percent AB_5_) and MFI (particle formation). Increasing the PS-80 concentration in the candidate formulation mitigated protein loss following freeze-thaw as measured by A280 using UV–Vis spectroscopy compared to the current formulation (Fig. 5A). No protein loss was observed in the candidate formulation with 0.1% PS-80, pH 7.4 formulation after 5 FT cycles. The HIC analysis showed no loss of native dmLT (AB_5_) for the candidate formulation with 0.1% v/v PS-80 while a substantial loss of native dmLT (∼19%) was observed in the current formulation after five F/T cycles (Fig. 5B). The size distribution and total number of sub-visible particles/aggregates formed during F/T in all the formulations was analyzed by MFI and is shown as a radar plot in Fig. 5C. The number of sub-visible particles increased slightly in the candidate formulation with 0.01% PS-80 but no change in the number of sub-visible particles was observed in the candidate formulation with 0.05% or 0.1% PS-80 after five FT cycles. The majority of sub-visible particles in all samples were 2–5 µm. In summary, increasing the PS-80 concentration from 0.01 to 0.1% v/v helped to minimize the protein loss and loss of the AB_5_ species, without causing any major change in the total number or distribution of sub-visible particles after 5 F/T cycles.

### 3.4 Summary of physicochemical stabilization of dmLT in candidate bulk formulation

The optimized candidate formulation for bulk dmLT which displayed increased thermal stability, reduced propensity to aggregate, robust freeze-thaw stability as well possession of acceptable solution tonicity was 50 mM sodium phosphate, 50 mM NaCl, 10% w/v sucrose, 5 mM methionine, 0.1% v/v PS-80, pH 7.4.

The new candidate formulation provided improved physical stability of dmLT, compared to the current formulation (used for lyophilization), upon agitation and F/T stresses as observed by the lower number of sub-visible particle formed and the lack of loss of protein concentration after five F/T cycles, respectively (Table 2). Although the aggregation propensity of dmLT upon thermal stress in the new candidate bulk formulation was slightly higher than in the current formulation (∼2 °C difference in OD350 values), given such high thermal stability values in either formulation (>70 °C), it is unlikely that the two formulation will differ in thermal stability when exposed to more moderate temperatures during accelerated and real time stability studies. Furthermore, no major differences were observed between dmLT in the two formulations in terms of relative AB_5_ ratio as measured by HIC and overall conformational stability (Tm values) as measured by DSC.

**Table 2:** Comparison of final candidate formulation vs current dmLT formulation in terms of relative percent AB_5_ as measured by HIC, solution osmolality as well as physicochemical stability (agitation, thermal, freeze-thaw, chemical and conformational stability) properties as measured by MFI, OD_350_ assay, UV visible spectroscopy, Intact MS and DSC, respectively.

Formulation	HIC	Osmolality	Agitation stability	Thermal stability (against aggregation)	F/T stability	Chemical stability glycation	Chemical stability oxidation	Conformational stability
	Relative% (AB_5_)	(mOSm)	MFI (ΔTotal particles_4h-0h_ > 2 µm, number/mL)	Temperature@OD_350_ = 0.1 unit (°C)	% Protein loss after 5FT	(Stressed at 40 °C for 7 days)	(Addition of 0, 1, 2.5 and 5 mM H_2_O_2_)	Tm values (DSC)
50 mM sodium phosphate, 50 mM NaCl, 10% w/v sucrose, 5 mM methionine, 0.1%v/v PS-80 pH 7.4 *(Candidate formulation)*	66 ± 1	500 ± 7	8.9 ± 2.9 x 10^2^	72.5 ± 0.2	0.0 ± 0.1	No change in relative abundance of the glycated B-chain nor formation of additional glycated peaks	No oxidation detected at 1 and 2.5 mM H_2_O_2_	T_m_1 = 50.9 ± 0.2 T_m_2 = 52.8 ± 0.1 T_m_3 = 79.3 ± 0.1 T_m_4 = 82.0 ± 0.1
42.7 mM sodium phosphate, 10.7 mM potassium phosphate, 82 mM NaCl, 5% lactose pH 7.4 *(Current formulation)*	66 ± 1	408 ± 3	275.2 ± 11.8 x 10^2^	74.4 ± 0.2	15.2 ± 0.3	Increased glycation and formation of additional glycated peaks	Oxidation detected at 1, 2.5 and 5 mM H_2_O_2_	T_m_1 = 51.3 ± 0.2 T_m_2 = 52.8 ± 0.1 T_m_3 = 79.5 ± 0.1 T_m_4 = 81.9 ± 0.1

**Supplementary Figure 2:**
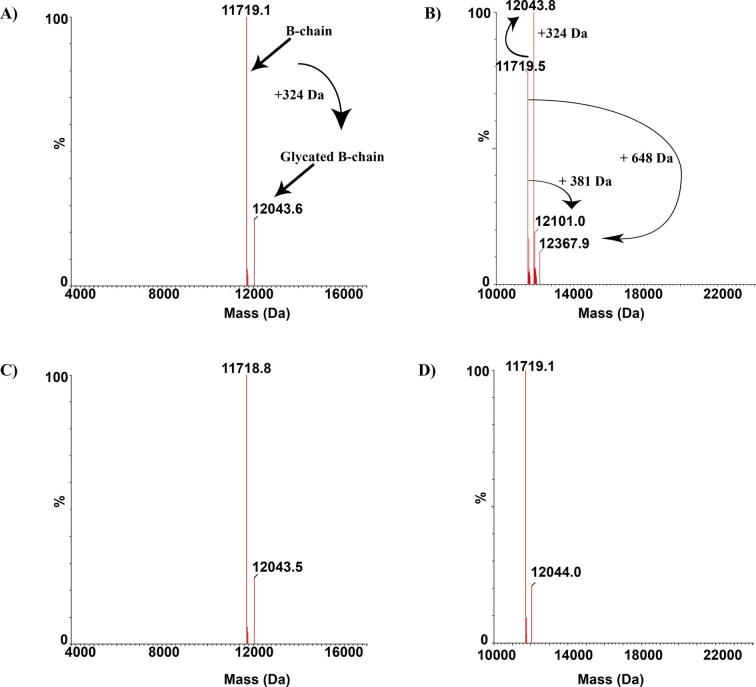


**Supplementary Figure 3:**
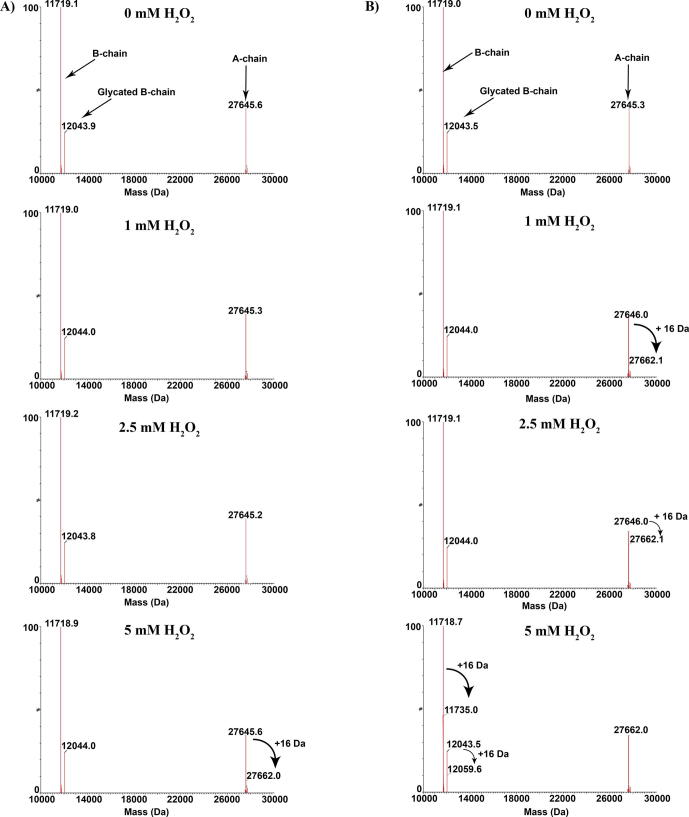


The new candidate bulk formulation also improved the chemical stability profile of dmLT, in terms of Lys glycation and Met oxidation, in comparison to dmLT in the current formulation (used for lyophilization). After incubation of dmLT at 40 °C for 7 days in the candidate formulation, no increase in relative abundance of the glycated B-chain, nor the formation of additional modified B-chain peaks (+381 Da and +648 Da mass increases), was observed by intact mass spectrometry (see Supplemental Fig. S2 and Supplemental Table S2). In contrast, dmLT in the current formulation under the same stress conditions showed increases in these chemically modified (glycated) species (see Supplemental Fig. S2 and Supplemental Table S2). Results of intact mass spectrometry analysis of dmLT in the two formulations exposed to different concentrations of hydrogen peroxide (0, 1, 2.5 and 5 mM) showed oxidized dmLT species (+16 Da) in the current formulation at 1, 2.5 and 5 mM H_2_O_2_ (see Supplemental Fig. S3 and Supplemental Table S3). However, no dmLT oxidation was detected in the candidate bulk formulation up to 2.5 mM H_2_O_2_, and thus, the new formulation appeared to be overall better than the current formulation in terms of protecting dmLT against oxidation stress (see Supplemental Fig. S3).

A comparative summary of the physicochemical properties of dmLT in the current (lyophilized and reconstituted) and new candidate formulation (bulk stored frozen) in terms of aggregation propensity due to thermal, agitation and F/T stresses, conformational stability as measured by DSC, chemical stability under forced oxidation and glycation conditions, AB5 ratio as measured by HIC, and solution osmolality are summarized in Table 2.

## 4 Discussion

Preformulation characterization and formulation development activities of vaccine candidates (both antigens and adjuvants) are critical activities for their clinical development and regulatory approval. A proper vaccine formulation should not only be stable (including the antigen and adjuvant are physically compatible), but the vaccine also must remain potent and the adjuvant must enhance the immunogenicity of the vaccine antigen. It is therefore imperative to assess and identify the structural integrity as well as physicochemical degradation pathways of antigen and adjuvant as part of formulation development. A rational design of stable formulation conditions is possible based on understanding the causes and mechanisms of vaccine antigen and adjuvant stability (and interactions), to maintain vaccine potency throughout the shelf life at the defined storage temperature(s). In this work, we have developed an improved bulk formulation of the dmLT adjuvant with the goal of providing long term frozen storage as well as flexibility for potential use with different vaccine antigens administered by various routes (e.g., injection, oral, nasal, etc.). The systematic formulation strategy described in this paper provides an overview of the physicochemical stability of dmLT in the presence of different excipients and not only helps to elucidate degradation pathways, but provides strategies for dmLT stabilization within a pharmaceutical dosage form.

The major physicochemical instabilities identified with the current formulation of dmLT were glycation and oxidation of specific Lys and Met residues, respectively, as well as protein aggregation induced by agitation and thermal stress (Toprani et al., 2017, submitted). Physical instability due to either heat and/or freeze stress of antigens or adjuvants can be a major cause of potency loss in various vaccines during manufacturing, storage and administration even within the vaccine cold chain [9], [24]. The lack of integrity of the vaccine cold-chain exposes vaccines to temperature fluctuations (either accidental heat exposure or freezing) during shipping, handling, distribution and administration to patients [9], [12], [24]. This puts an additional requirement on vaccine manufacturers to develop more thermostable vaccines or vaccine formulations to make their products more widely available in global markets.

With the aim to develop a more stable bulk dmLT formulation that does not require lyophilization, this work involved identifying pharmaceutical excipients that enhanced dmLT stability (i.e., minimized aggregation during thermal and agitation stresses). Sucrose and phosphate were identified as stabilizers that increased dmLT thermal stability against aggregation and limited its aggregation. Sucrose is a widely used pharmaceutical excipient that stabilizes biomolecules, including antibodies, protein drugs and vaccines [25], [26], [27] by offering protection against elevated temperatures as well as freezing stress via preferential exclusion mechanisms [28], [29]. Additionally, its non-reducing nature doesn’t cause glycation in proteins unless exposed to very low pH or high temperatures [23]. In fact, our results demonstrated that dmLT in the candidate formulation (containing sucrose) showed essentially no Lys glycation formation during elevated temperature studies compared to dmLT in the current formulation (containing lactose) under the same conditions which displayed increased formation of glycated protein.

The investigation of the effect of phosphate on the thermal stability of dmLT at two pH conditions revealed that relatively higher concentrations (50 mM) of phosphate at pH 7.4 showed an increased thermal stability of dmLT compared to pH 6.0. At pH 7.4, a potential reason for the increased thermal stability (in terms of aggregation propensity) of dmLT under higher phosphate concentrations can probably be attributed to phosphate binding or intermolecular electrostatic effects. Proteins are net negatively charged above their isoelectric point (pI) and net positively charged below its pI [30]. The theoretical pI of the A-subunit of dmLT based on its amino acid sequence is ∼ 6.48. In the pH 7.4 formulation, above the A-subunit’s pI, the A-subunit is expected to have a net negative charge with a smaller proportion of positive charges. Addition of increased amounts of phosphate anions can effectively shield positive charges on the protein surfaces. This would cause an increase in intermolecular charge-charge repulsions between dmLT molecules making it less favorable for the two molecules to interact (aggregate) with an increase in stability as was observed in this work by OD_350_ thermal melt results (see Fig. 4A). In contrast, at pH 6.0 (closer to the theoretical A chain pI = 6.48), the A-subunit of dmLT has anisotropic charge distribution which may give rise to dipoles which in turn could make attractive forces between dmLT molecules to dominate, making aggregation more favorable. Multiple studies on different proteins have also demonstrated the role of increasing repulsive charge-charge interactions in stabilizing protein solutions (i.e., colloidal stability) and thereby reducing/preventing protein aggregation [31], [32], [33].

Agitation is a common physical stress experienced during manufacturing, shipping and handling of protein based drugs and vaccines [34]. It can cause protein structural alterations at the air-liquid interface leading to formation of nucleating aggregated species in the bulk solution causing more extensive aggregation in the bulk formulation [34], [35]. To protect dmLT against aggregation caused by agitation stress, polysorbate-80 was identified as a stabilizing excipient. PS-80 mitigated aggregate/particle formation in dmLT during agitation by presumably outcompeting proteins for the air-water interface and thus inhibiting surface adsorption and structural alterations of proteins due to the hydrophobic nature of air water interfaces. PS-80 is a non-ionic surfactant widely used with protein drugs and some vaccines to prevent aggregation against a number of interfacial stresses including agitation and freeze thaw [36], [37]. PS-80 is also known to protect freezing induced perturbations in protein conformation leading to protein aggregation and reduce the unfolding of proteins at ice-water interfaces [38]. These effects are presumably responsible for the stabilization of dmLT (no loss of protein) observed in this work during multiple freeze-thaw cycles.

Protein oxidation is a major chemical degradation pathway for protein drugs [39]. Oxidation can be induced during manufacturing by trace metal ions leached from equipment or exposure of proteins to light and oxygen from the surrounding air. Certain excipients such as non-ionic surfactants can also form peroxides which are a major catalyst of protein oxidation [39]. Oxidation can affect pharmaceutical properties of proteins, including solubility, conformation, biological activity and shelf-life [39]. There are many potential ways to protect against oxidation such as site-directed mutagenesis to remove labile amino acid residues, development of solid state vs liquid formulations, or addition of excipients with anti-oxidant properties such as methionine in the formulation. To protect dmLT against oxidation, the amino acid methionine was added in the formulation since Met can act as an oxidation scavenger. Lam et al. [40] have reported that addition of methionine to a formulation of rhuMAb HER2 protected it against temperature and light induced oxidation. The authors concluded that exogenous methionine can act as an antioxidant by either inhibiting the free radical chain reactions during oxidation or by competing with the endogenous methionine residues in the protein for reactions with hydroxyl radicals. In fact, our results demonstrated that dmLT in the candidate formulation containing methionine showed reduced levels of oxidation of Met residues in the dmLT protein compared to dmLT in the current formulation under the same forced oxidation conditions.

One important aspect of vaccine formulation development is to permit stable, long term frozen storage of the bulk vaccine antigen (or in this case, dmLT adjuvant). Freezing protein bulk offers several potential advantages such as increased protein storage stability and shelf life, a decreased risk of microbial growth, elimination of agitation during transport and flexibility in subsequent fill-finish manufacturing [41]. For example, if the bulk protein vaccine antigen (or in this case, dmLT adjuvant) can withstand freeze-thaw cycle, it may be frozen at the bulk manufacturing site until transport to the fill finish site, and the subsequent drug product could potentially be stored and shipped frozen to a clinical site. However, freeze-thaw stress can negatively impact the structural integrity and potency of protein drugs and vaccine [38], [42], [43], including the freeze-thaw induced aggregation of aluminum salt adjuvants used in recombinant protein vaccines [44], [45] leading to loss of biologic activity [46]. Freeze-thaw studies provide data on the impact of freezing on structure and conformation of protein adjuvants and antigens. Currently, majority of vaccines in preclinical or clinical development are based on recombinant proteins which require an adjuvant to increase their potency [47], [48]. Hence, if the vaccine requires freezing for storage with dmLT as an adjuvant, then both the antigen and dmLT adjuvant should be able to withstand freeze-thaw effects. The freeze-thaw study here showed that dmLT in the newly developed formulation was freeze-thaw stable for up to five FT cycles. Neither aggregation nor loss of protein was observed after five freeze thaw cycles.

A combination of the key excipients such as phosphate ion, sucrose, methionine and polysorbate 80 showed protection of dmLT against thermal, agitation, and freeze-thaw stresses. This was due to inhibition of aggregation as well as minimization of chemical alterations including Lys glycation and Met oxidation. The potential benefits of this candidate formulation can not only be in protecting and stabilizing the dmLT protein adjuvant against different stresses, but also offering flexibility in terms of combining dmLT with different antigens. Furthermore, an additional benefit of the candidate bulk formulation of dmLT offers is its pH (pH 7.4) since formulations at physiological pH may minimize aggregation or precipitation caused by transitioning from formulation pH to physiological pH conditions [49]. Finally, the replacement of lactose with sucrose in the new candidate formulation should eliminate Lys glycation of dmLT with a simultaneous increase in thermal and freeze-thaw stability of dmLT.

In conclusion, this study provides development of a new candidate bulk formulation of dmLT adjuvant by better understanding the protein’s key structural features, identifying its physicochemical degradant pathways, and identifying stabilizing excipients to minimize protein instability by the use of appropriate stability indicating analytical assays. The dmLT protein adjuvant has shown potential to function as a mucosal adjuvant with a wide variety of antigens in both animal and human studies by a variety of administration routes [50]. For further pharmaceutical development of dmLT in the new candidate bulk formulation, long term bulk storage stability studies, and compatibility testing with different vaccine antigens in a final drug product will be necessary. In addition, immunogenicity studies in animal models of dmLT in candidate formulations along with a co-administered antigen are also suggested. For example, the adjuvanticity and antigenicity of dmLT with a tetanus toxoid antigen has been evaluated in mice by measuring serum anti-antigen IgG and serum anti-LT IgG levels, respectively [17].

## References

[1] Reed S.G., Bertholet S., Coler R.N., Friede M. (2009). New horizons in adjuvants for vaccine development. Trends Immunol.

[2] Petrovsky N., Aguilar J.C. (2004). Vaccine adjuvants: current state and future trends. Immunol Cell Biol.

[3] Coffman R.L., Sher A., Seder R.A. (2010). Vaccine adjuvants: putting innate immunity to work. Immunity.

[4] Brito L.A., Malyala P., O’Hagan D.T. (2013). Vaccine adjuvant formulations: a pharmaceutical perspective. Semin Immunol.

[5] Morefield G.L. (2011). A rational, systematic approach for the development of vaccine formulations. The AAPS J.

[6] O’Hagan D.T., Fox C.B. (2015). New generation adjuvants – from empiricism to rational design. Vaccine.

[7] Dey A.K., Malyala P., Singh M. (2014). Physicochemical and functional characterization of vaccine antigens and adjuvants. Expert Rev Vaccines.

[8] Frokjaer S., Otzen D.E. (2005). Protein drug stability: a formulation challenge. Nat Rev Drug Discov.

[9] Kartoglu U., Milstien J. (2014). Tools and approaches to ensure quality of vaccines throughout the cold chain. Expert Rev Vaccines.

[10] Vázquez-Rey M., Lang D.A. (2011). Aggregates in monoclonal antibody manufacturing processes. Biotechnol Bioeng.

[11] Wang W., Roberts Christopher John (2010). Aggregation of therapeutic proteins.

[12] Kumru O.S., Joshi S.B., Smith D.E., Middaugh C.R., Prusik T., Volkin D.B. (2014). Vaccine instability in the cold chain: mechanisms, analysis and formulation strategies. Biologicals.

[13] Kamerzell T.J., Esfandiary R., Joshi S.B., Middaugh C.R., Volkin D.B. (2011). Protein–excipient interactions: mechanisms and biophysical characterization applied to protein formulation development. Adv Drug Deliv Rev.

[14] Parkins D.A., Lashmar U.T. (2000). The formulation of biopharmaceutical products. Pharm Sci Technol Today.

[15] Ohtake S., Kita Y., Arakawa T. (2011). Interactions of formulation excipients with proteins in solution and in the dried state. Adv Drug Deliv Rev.

[16] Capelle M.A.H., Gurny R., Arvinte T. (2007). High throughput screening of protein formulation stability: practical considerations. Eur J Pharm Biopharm.

[17] Norton E.B., Lawson L.B., Freytag L.C., Clements J.D. (2011). Characterization of a mutant Escherichia coli heat-labile toxin, LT(R192G/L211A), as a safe and effective oral adjuvant. Clin Vaccine Immunol: CVI.

[18] Piedmonte D.M., Summers C., McAuley A., Karamujic L., Ratnaswamy G. (2007). Sorbitol crystallization can lead to protein aggregation in frozen protein formulations. Pharm Res.

[19] Piedmonte D.M., Hair A., Baker P., Brych L., Nagapudi K., Lin H. (2015). Sorbitol crystallization-induced aggregation in frozen mAb formulations. J Pharm Sci.

[20] Meyer J.D., Nayar R., Manning M.C. (2009). Impact of bulking agents on the stability of a lyophilized monoclonal antibody. Eur J Pharm Sci.

[21] Han Y., Jin B.-S., Lee S.-B., Sohn Y., Joung J.-W., Lee J.-H. (2007). Effects of sugar additives on protein stability of recombinant human serum albumin during lyophilization and storage. Arch Pharmacal Res.

[22] Morgan F., Léonil J., Mollé D., Bouhallab S. (1999). Modification of bovine β-lactoglobulin by glycation in a powdered state or in an aqueous solution: effect on association behavior and protein conformation. J Agric Food Chem.

[23] Fischer S., Hoernschemeyer J., Mahler H.-C. (2008). Glycation during storage and administration of monoclonal antibody formulations. Eur J Pharm Biopharm.

[24] Chen D.D. (2009). Opportunities and challenges of developing thermostable vaccines. Expert Rev Vaccines.

[25] Prevention CfDCa. Vaccine excipient & media summary. Excipients included in U.S. vaccines, by vaccine http://www.cdc.gov/vaccines/pubs/pinkbook/downloads/appendices/B/excipient-table-2.pdf [accessed 23.07.16].

[26] Kendrick B.S., Chang B.S., Arakawa T., Peterson B., Randolph T.W., Manning M.C. (1997). Preferential exclusion of sucrose from recombinant interleukin-1 receptor antagonist: Role in restricted conformational mobility and compaction of native state. Proc Natl Acad Sci USA.

[27] Carpenter J.F., Pikal M.J., Chang B.S., Randolph T.W. (1997). Rational design of stable lyophilized protein formulations: some practical advice. Pharm Res.

[28] Lee J.C., Timasheff S.N. (1981). The stabilization of proteins by sucrose. J Biol Chem.

[29] Kim Y.-S., Jones L.S., Dong A., Kendrick B.S., Chang B.S., Manning M.C. (2003). Effects of sucrose on conformational equilibria and fluctuations within the native-state ensemble of proteins. Protein Sci: A Publ Protein Soc.

[30] Dill K.A. (1990). Dominant forces in protein folding. Biochemistry.

[31] Chi E.Y.E.Y. (2003). Roles of conformational stability and colloidal stability in the aggregation of recombinant human granulocyte colony-stimulating factor. Protein Sci.

[32] Chi E.Y., Krishnan S., Randolph T.W., Carpenter J.F. (2003). Physical stability of proteins in aqueous solution: mechanism and driving forces in nonnative protein aggregation. Pharm Res.

[33] Tsai A.M.A. (1998). II. Electrostatic effect in the aggregation of heat-denatured RNase A and implications for protein additive design. Biotechnol Bioeng.

[34] Krielgaard L., Jones L.S., Randolph T.W., Frokjaer S., Flink J.M., Manning M.C. (1998). Effect of tween 20 on freeze-thawing- and agitation-induced aggregation of recombinant human factor XIII. J Pharm Sci.

[35] Mahler H.-C., Fischer S., Randolph T.W., Carpenter J.F. (2010). Protein aggregation and particle formation: effects of formulation, interfaces, and drug product manufacturing operations.

[36] Lee H.J., McAuley A., Schilke K.F., McGuire J. (2011). Molecular origins of surfactant-mediated stabilization of protein drugs. Adv Drug Deliv Rev.

[37] Kerwin B.A. (2008). Polysorbates 20 and 80 used in the formulation of protein biotherapeutics: structure and degradation pathways. J Pharm Sci.

[38] Chang B.S., Kendrick B.S., Carpenter J.F. (1996). Surface-induced denaturation of proteins during freezing and its inhibition by surfactants. J Pharm Sci.

[39] Li S., Schöneich C., Borchardt R.T. (1995). Chemical instability of protein pharmaceuticals: mechanisms of oxidation and strategies for stabilization. Biotechnol Bioeng.

[40] Lam X.M., Yang J.Y., Cleland J.L. (1997). Antioxidants for prevention of methionine oxidation in recombinant monoclonal antibody HER2. J Pharm Sci.

[41] Singh S.K., Kolhe P., Mehta A.P., Chico S.C., Lary A.L., Huang M. (2011). Frozen state storage instability of a monoclonal antibody: aggregation as a consequence of trehalose crystallization and protein unfolding. Pharm Res.

[42] Cold Denaturation of Protein (1990). Crit Rev Biochem Mol Biol.

[43] Jaenicke R., Heber U., Franks F., Chapman D., Griffin M.C.A., Hvidt A. (1990). Protein structure and function at low temperatures [and discussion]. Philos Trans R Soc B: Biol Sci.

[44] Clausi A.L., Morin A., Carpenter J.F., Randolph T.W. (2009). Influence of protein conformation and adjuvant aggregation on the effectiveness of aluminum hydroxide adjuvant in a model alkaline phosphatase vaccine. J Pharm Sci.

[45] Salnikova M.S., Davis H., Mensch C., Celano L., Thiriot D.S. (2012). Influence of formulation pH and suspension state on freezing-induced agglomeration of aluminum adjuvants. J Pharm Sci.

[46] Bhatnagar B.S. (2007). Protein stability during freezing: separation of stresses and mechanisms of protein stabilization. Pharm Dev Technol.

[47] Nascimento I.P., Leite L.C.C. (2012). Recombinant vaccines and the development of new vaccine strategies. Braz J Med Biol Res.

[48] Perrie Y., Mohammed A.R., Kirby D.J., McNeil S.E., Bramwell V.W. (2008). Vaccine adjuvant systems: enhancing the efficacy of sub-unit protein antigens. Int J Pharm.

[49] Kolhe P., Shah M., Rathore N. (2013). Molecule and manufacturability assessment leading to robust commercial formulation for therapeutic proteins.

[50] Bourgeois A.L., Wierzba T.F., Walker R.I. (2016). Status of vaccine research and development for enterotoxigenic Escherichia coli. Vaccine.

