# Supplementary material for: Development of a candidate stabilizing formulation for bulk storage of a double mutant heat labile toxin (dmLT) protein based adjuvant
Source: Vaccine. 2017 Oct 4;35(41):5471–80. doi: 10.1016/j.vaccine.2017.03.101 (PMC5628956; doi:10.1016/j.vaccine.2017.03.101)
Supplement: Supplementary data 1 [file mmc1.docx]

**SUPPLEMENTAL SECTION**

**Development of a Candidate Stabilizing Formulation for Bulk Storage of a Double Mutant Heat Labile Toxin (dmLT) Protein Adjuvant**

Vishal Toprani^a^, Neha Sahni^a^, John M. Hickey^a^, George A. Robertson^b^, C. Russell Middaugh^a^, Sangeeta B. Joshi^a^, and David B. Volkin^a^

^a^ Department of Pharmaceutical Chemistry, Macromolecule and Vaccine Stabilization Center, University of Kansas, 2030 Becker Drive, Lawrence, Kansas 66047, USA

^b^ The Center for Vaccine Innovation and Access, PATH, 455 Massachusetts Ave NW Suite 1000, Washington, DC 20001

**Correspondence to:** David B. Volkin: 2030 Becker Drive, Lawrence, KS 66047, Email: volkin@ku.edu; Phone: 785-864-6262; Fax: 785-864-5736

**SUPPLEMENTAL METHODS SECTION:**

**Thermal and agitation stress studies of dmLT:**

For excipient screening studies as a function of both a thermal stress and shake-stress, lyophilized dmLT samples (BPR # 1037.00) were reconstituted in HPLC grade water at room temperature. Reconstitution time was less than 1 min. Multiple vials of reconstituted dmLT were then pooled and the protein concentration was determined (~1 mg/mL). The samples were centrifuged for 15 min at 5,000 rpm and the protein concentration was determined (~0.9 mg/ml). The dmLT protein was dialyzed in Slide-A-Lyzer Mini Dialysis Devices (Product #88403, Thermo Scientific, Rockford, IL) with a 3,500 Da molecular weight cutoff and a 2 mL capacity against various base buffers, (10 mM Sodium Phosphate, containing 50 or 150 mM NaCl, pH 6.0) or (50 mM sodium phosphate, 50 mM NaCl, pH 7.4) overnight at 4°C with three buffer changes at three hr. intervals, and incubation overnight before dialysate recovery. The protein concentration in the dialysate was determined both pre and post centrifugation (13,000 rpm for 5 min). Excipients were prepared as concentrated solutions in the indicated base buffers. Each of the excipients, dmLT, and additional base buffer were then combined to achieve the concentrations of excipients indicated in the text and a final concentration of 0.15 mg/mL dmLT. For shake stress studies, 2 mL Fiolax clear, Schott (Lebanon, PA) glass vials were filled with 0.6 mL of 0.15 mg/mL dmLT and stoppered (Cat#10122128, West Pharmaceutical, PA). The vials were then shaken sideways at 300 RPM for 4 hr. at room-temperature. Additionally, control vials were filled with 0.6 mL of the each buffer without dmLT and shaken under similar conditions. All analytical techniques mentioned below were performed in triplicate on control/non-shake samples and the 4 hr. shaking stress samples.

**Freeze-thaw studies of dmLT:**

The dmLT vials were reconstituted with water and dialyzed against the indicated base buffers (see above) in Slide-A-Lyzer Mini Dialysis Devices (Product #88403, Thermo Scientific, Rockford, IL) with a 3,500 Da molecular weight cutoff overnight at 4°C with three buffer changes at three hr. intervals, and incubation overnight before dialysate recovery. Excipients were prepared as concentrated solutions in indicated base buffer. Each of the excipients, dmLT, and additional base buffer were combined to achieve the final formulations as indicated in text. Then, 1.1 mL of dmLT at 0.4 mg/mL was filled in 3 mL Fiolax clear, Schott (Lebanon, PA) glass vials and then frozen at -80^o^C for 24 hrs. The vials were thawed at room temperature for ~20 min for complete thawing. Samples were then frozen again at -80^o^C and the freeze-thaw (F/T) cycles were repeated five times. Samples were analyzed at 0, 1 and 5 F/T cycles using UV-Visible absorption spectroscopy, hydrophobic interaction chromatography (see Toprani et.al, 2016 in this current issue) and MFI as described below.

## UV-Visible Spectroscopy

The UV-Visible absorption spectra of dmLT were recorded with an Agilent 8453 UV-Visible spectrophotometer (Palo Alto, CA) equipped with a peltier temperature controller. Spectra were collected from 190-1100 nm at an experimental resolution of 1 nm in 1 cm path length quartz cuvette. The protein concentration was calculated based on the reported extinction coefficient (0.1% w/v solution) of 1.14 mg/ml^-1^ cm^-1^. Samples were measured before and after centrifugation (5000 rpm for 15 min, Thermo Scientific Sorvall Centrifuge, Waltham, MA). The instrument was first blanked using each respective formulation buffer prior to measuring solutions containing dmLT. The UV-Visible absorbance spectra were corrected for light scattering using a technique included in the manufacturer’s data analysis software (Chemstation UV-Vis analysis software, Agilent Technologies): first, the spectra data, where the optical density values are only due to light scattering (350-400 nm), are fitted to an equation, and then this curve is extrapolated across the entire protein spectrum, and then subtracted from the original spectrum, to produce the light-scatter corrected absorbance spectra. The optical density value at 350 nm (OD350) was also recorded from the uncorrected spectra. A similar procedure was followed for measuring protein concentration after dialysis, both before and after centrifugation.

## OD_350_ measurements

Thermal stress measurements were performed using a Cary 100 UV-Visible spectrophotometer (Varian medical Systems, Inc., Palo Alto, California) equipped with a 12 cell holder with a peltier type temperature controller. Samples contained 0.15 mg/ml protein with a total volume of 225 µl in 1 cm path length quartz cells. Optical density at 350 nm (OD350) was monitored as the temperature was raised in increments of 1.25˚C from 10 to 90˚C with a heating rate of 60˚C/h. Protein samples were run in triplicate and corresponding buffer blanks were run and subtracted from each sample. The OD350 value was plotted against temperature, and the temperature at which the OD350 value reached an optical density value of 0.1 was determined.

## Micro-Flow Imaging (MFI)

The total number and distribution of sub-visible particles in the range of 2 µm to 100 µm were examined using a MFI DPA-4200 (Protein Simple, Santa Clara, CA) system with a 100 µm silane coated flow cell. Measurements were made in triplicate at ambient temperature for all samples. Illumination was optimized using particle free water prior to all measurements.

**Hydrophobic Interaction Chromatography (HIC):**

A Shimadzu Prominence UFLC HPLC system equipped with a diode array detector was used. 20 μg of protein was injected onto a TSKgel Butyl-NPR column (4.6 x 100mm, 2.5µm TOSOH Biosciences P/N 42168) for each run, and the experiment was performed in triplicate. The mobile phases consisted of (A) 2M ammonium sulfate, 20 mM sodium phosphate, pH 6.8; (B) 20 mM sodium phosphate, pH 6.8. The columns were equilibrated with mobile phase A (20 mM sodium phosphate, 2 M ammonium sulfate, pH 6.8) prior to sample injection. A flow rate of 0.7 mL/minute was used with a 60 minute run time. Chromatographic separation was then conducted in a step wise gradient of 0-5 min (0% B), 5-35 min (0$\to$60% B), 35-40 min (60$\to$100% B), 40-45 min (100% B), and 45-60 min (0% B). Protein peaks were monitored using the absorbance signal at 214 nm. LC solutions software (Shimadzu) was used for data analysis.

**Differential Scanning Calorimetry**

DSC thermograms for dmLT were collected with a Microcal VP-DSC capillary cell microcalorimeter (MicroCal/GE Health Sciences, Pittsburgh, PA). Thermograms were recorded from 10 to 100 °C at a scan rate of 1 °C/min. The concentration of dmLT was 0.4 mg/ml and the experiment was performed in triplicate. A buffer baseline was subtracted from each protein thermogram and the data were normalized to molar heat capacity using Microcal DSC software in Origin 7.0. The peaks were fitted using mathematical model fit in Origin 7.0 to calculate the values of T_onset_ and T_m_.

**Intact Mass Spectrometry-**

ESI spectra of dmLT were acquired on a SYNAPT G2 hybrid quadrupole / ion mobility / Tof mass spectrometer (Waters Corp., Milford, MA). The instrument was operated in a sensitivity mode with all lenses optimized on the MH+ ion from the control Leucine Enkephalin. The sample cone voltage was 40eV. Argon was admitted to the trap cell that was operated at 4eV for maximum transmission. Spectra were acquired at 9091 Hz pusher frequency covering the mass range from 100 to 3000 u and accumulating data for 2 seconds per cycle. Time to mass calibration was made with NaI cluster ions acquired under the same conditions. Mass spectra of [Glu^1^]-Fibrinopeptide B were acquired in parallel scans and doubly charged ions at m/z 785.8426 were used as a lock mass reference.

Samples were desalted on a reversed phase PRP-1 column, 1 cm, 1 mm I.D. (Hamilton, 10 µm particles packed by hand) using a NanoAcquity chromatographic system (Waters Corporation). The mobile phase solvents were A (99.9% H_2_O, 0.1% formic acid) and B (99.9% acetonitrile, 0.1% formic acid). A short gradient was developed from 1 to 70% B in 4 min with a flow rate of 20 µL/min. MassLynx 4.1 software (Waters Corporation) was used to collect the data and to deconvolute the protein spectra for molecular weight determination.

**Chemical Stability Studies with dmLT (Oxidation and Glycation):**

Forced glycation studies were carried out with dmLT in the two formulations namely:

1. 42.7 mM Sodium Phosphate, 10.7 mM Potassium Phosphate, 82 mM NaCl, 5% Lactose, pH 7.4 (Current formulation)
2. 50 mM Sodium phosphate, 50 mM NaCl, 10% sucrose, 5 mM methionine, 0.1% PS-80, pH 7.4 (Candidate Formulation)

The dmLT samples in the two different formulations (at 0.4 mg/mL) were incubated in triplicate at 40^o^C for 7 days. On days 0 and 7, the samples were subjected to intact mass analysis

For forced oxidation studies, dmLT samples at 0.4 mg/mL in the current and candidate formulation was oxidized in triplicate for four hours at 37^o^C with each of the following H_2_O_2_ concentrations: 0, 1, 2.5 and 5 mM. Reactions were quenched with 70 mM of D-Met and all samples were subjected to intact mass analysis.

.
