# Supplementary material for: Development of a candidate stabilizing formulation for bulk storage of a double mutant heat labile toxin (dmLT) protein based adjuvant
Source: Vaccine. 2017 Oct 4;35(41):5471–80. doi: 10.1016/j.vaccine.2017.03.101 (PMC5628956; doi:10.1016/j.vaccine.2017.03.101)
Supplement: Supplementary data 2 [file mmc2.docx]

**Supplemental Table S1**. List of pharmaceutical excipients selected for further optimization for formulation design of dmLT. Excipients with a (+) sign indicate the excipient when added to base buffer (10 mM sodium phosphate, 150 mM NaCl, pH 6.0), showed a stabilizing effect on inhibiting dmLT aggregation as measured by OD350 assay for thermal stability and as measured by MFI for agitation stability. The excipients highlighted in yellow in Supplemental Table S1 were chosen for further optimization based on their most effective overall stabilizing effect with dmLT.

| **Excipient Category** | **Excipient** | **Thermal Stress** | **Agitation stress** |
| --- | --- | --- | --- |
| **Salts/Buffers** | Sodium acetate | + | + |
|  | Sodium sulfate (salt) | + | + |
|  | Sodium succinate | + |  |
| **Proteins** | Human Albumin |  | + |
|  | Hydrolysed Gelatin |  | + |
|  | Protamine sulfate |  | + |
| **Amino acids and Miscellaneous** | Arginine |  | + |
|  | Aspartic acid | + | + |
|  | Glutamic acid | + | + |
|  | Glycine |  | + |
|  | Histidine |  | + |
|  | Isoleucine |  | + |
|  | Lactic acid |  | + |
|  | Lysine |  | + |
|  | Methionine | + | + |
|  | Proline | + | + |
|  | Urea |  | + |
|  | Arginine + Glutamic acid |  | + |
|  | Arginine + Glutamic acid + Isoleucine |  |  |
| **Metal ions/Chelators/Reducing agents** | Calcium chloride |  | + |
|  | DTT |  | + |
|  | EDTA |  | + |
|  | Magnesium chloride |  | + |
| **Detergents** | Brij 35 |  | + |
|  | Poloxamer 188 (Pluronic F-68) |  | + |
|  | Polysorbate 20 |  | + |
|  | Polysorbate 80 |  | + |
|  | Triton X-100 |  |  |
| **Carbohydrates** | Lactose | + | + |
|  | Sucrose | + | + |
|  | Trehalose | + |  |
| **Polyols** | Glycerol | + | + |
|  | Mannitol | + | + |
|  | Sorbitol | + | + |
| **Cyclodextrins** | 2-OH propyl b-CD |  | + |
| **Polymers/Osmolyte/Polyions** | Carboxymethyl cellulose |  | + |
|  | Dextran sulfate |  | + |
|  | Dextran 40 |  | + |
|  | PEG-3350 |  | + |
|  | Sodium Hyaluronate |  | + |

**Supplemental Table S2.** A summary of the intact protein mass spectrometry analysis of dmLT in the candidate vs current formulation after forced glycation studies. Data represent the average and standard deviation for n=3 replicates.

| **Time** | **dmLT in Candidate Formulation**  **MW(Da)** | **dmLT in**  **Current Formulation**  **MW(Da)** |
| --- | --- | --- |
| Day = 0 | A chain = 27645.6 + 0.4  B-chain =11718.8 + 0.1  Glycated B-chain = 12043.3 + 0.3 | A chain = 27645.5 + 0.2  B-chain =11718.9 + 0.2  Glycated B-chain = 12043.5 + 0.1 |
| Day = 7 | A chain = 27646.1 + 0.4  B-chain =11719.2 + 0.1  Glycated B-chain = 12044.0 + 0.0 | A chain =27646.1 + 0.2  B-chain =11719.6 + 0.1  Glycated B-chain = 12043.8 + 0.1  12101.2 + 0.2  12367.9 + 0.1 |

**Supplemental Table S3.** A summary of the intact protein mass spectrometry analysis of dmLT in the candidate vs current formulation after forced oxidation studies. Data represent the average and standard deviation for n=3 replicates. NA is not applicable.

| **H_2_O_2_ (mM)** | **Subunit of dmLT** | **dmLT in Candidate Formulation**  **MW (Da)** | **dmLT in**  **Current Formulation**  **MW (Da)** |
| --- | --- | --- | --- |
| 0 | A-chain  B-chain  Glycated B-chain  Oxidized A-chain (+16 Da)  Oxidized B-chain (+16 Da)  Oxidized glycated B-chain (+16 Da) | 27645.6 + 0.4  11719.2 + 0.2  12043.9 + 0.3  NA  NA  NA | 27645.3 + 0.3  11719.0 + 0.2  12043.5 + 0.1  NA  NA  NA |
| 1 | A-chain  B-chain  Glycated B-chain  Oxidized A-chain (+16 Da)  Oxidized B-chain (+16 Da)  Oxidized glycated B-chain (+16 Da) | 27645.3 + 0.2  11719.0 + 0.1  12044.0 + 0.0  NA  NA  NA | 27646.0 + 0.2  11719.1 + 0.1  12044.0 + 0.1  27662.1+ 0.2  NA  NA |
| 2.5 | A-chain  B-chain  Glycated B-chain  Oxidized A-chain (+16 Da)  Oxidized B-chain (+16 Da)  Oxidized glycated B-chain (+16 Da) | 27645.2 + 0.3  11719.2 + 0.2  12043.8 + 0.1  NA  NA  NA | 27646.0 + 0.3  11719.1 + 0.1  12044.0 + 0.1  27662.1+ 0.1  NA  NA |
| 5 | A-chain  B-chain  Glycated B-chain  Oxidized A-chain (+16 Da)  Oxidized B-chain (+16 Da)  Oxidized glycated B-chain (+16 Da) | 27645.6 + 0.3  11718.9 + 0.2  12043.8 + 0.1  27662.0 + 0.3  NA  NA | NA  11718.7+ 0.2  12043.5 + 0.1  27662.1 + 0.2  11735.0+ 0.4  12059.7 + 0.2 |
