# Supplementary material for: Development of a candidate stabilizing formulation for bulk storage of a double mutant heat labile toxin (dmLT) protein based adjuvant
Source: Vaccine. 2017 Oct 4;35(41):5471–80. doi: 10.1016/j.vaccine.2017.03.101 (PMC5628956; doi:10.1016/j.vaccine.2017.03.101)
Supplement: Supplementary data 3 [file mmc3.docx]

**Supplemental Figure S1.** Effect of freeze-thaw (0, 1 and 5 freeze-thaw cycles) on stability of dmLT in two candidate formulations vs current formulation. (A) Absorbance at 280 nm showing protein loss with increasing freeze thaw cycles, (B) radar plot analysis of the number and size distribution of sub-visible particles formed upon freeze-thaw as measured by MFI. The dmLT protein concentration was 0.4 mg/mL in the three formulations namely candidate formulation F1- (10 mM phosphate, 50 mM NaCl, 10% w/v sucrose, 5 mM methionine, 0.01% v/v PS-80 pH 6.0), candidate Formulation F2- (50 mM phosphate, 50 mM NaCl, 10% w/v sucrose, 5 mM methionine, 0.01% v/v PS-80, pH 7.4) and Formulation F3- Current dmLT formulation, (42.7 mM sodium phosphate, 10.7 mM potassium phosphate, 82 mM NaCl, 5% lactose , pH 7.4). Error bars indicate standard deviation of triplicate samples.

**Supplemental Figure S2.** Forced glycation studies of dmLT formulated in candidate vs current formulations. (A and B). Representative intact protein mass spectrometry analysis of B-chain of dmLT in current formulation sample at time zero and after incubation at 40 ^o^C for 7 days, respectively, showing an increase in glycation of the B-chain as well as formation of additional glycated B-chain products (+381 and +648 Da), and (C and D) representative intact protein mass spectrometry analysis of B-chain of dmLT in new candidate formulation at time zero and after incubation at 40 ^o^C for 7 days, respectively.

**Supplemental Figure S3.** Forced oxidation studies of dmLT in candidate vs current formulation. A) Representative intact protein mass spectrometry analysis of dmLT in candidate formulation incubated for four hours at 37^o^C with 0, 1, 2.5 and 5 mM hydrogen peroxide, and B) representative intact mass spectrometry analysis of dmLT in the current formulation incubated under the same stress conditions.
